# Prothrombin time is predictive of low plasma prothrombin concentration and clinical outcome in patients with trauma hemorrhage: analyses of prospective observational cohort studies

**DOI:** 10.1186/s13049-016-0332-2

**Published:** 2017-03-14

**Authors:** Clare A. Balendran, Ann Lövgren, Kenny M. Hansson, Karin Nelander, Marita Olsson, Karin J. Johansson, Karim Brohi, Dietmar Fries, Anders Berggren

**Affiliations:** 1 Personalised HealthCare and Biomarkers, Innovative Medicines and Early Development Biotech Unit, AstraZeneca, Pepparedsleden 1, Mölndal, 431 83 Sweden; 2 Cardiovascular and Metabolic Diseases, Innovative Medicines and Early Development Biotech Unit, AstraZeneca, Pepparedsleden 1, Mölndal, 431 83 Sweden; 3 Early Clinical Development, Innovative Medicines and Early Development Biotech Unit, AstraZeneca, Pepparedsleden 1, Mölndal, 431 83 Sweden; 4 Present address: Leaflet Biotech Consulting, Jungfrudansen 8, 171 56 Solna, Sweden; 5 Centre for Trauma Sciences, Barts and the London School of Medicine and Dentistry, Queen Mary University of London, London, UK; 6 Department of General and Surgical Critical Care Medicine, Innsbruck Medical University, Innsbruck, Austria

**Keywords:** Prothrombin, Coagulopathy, Severe bleeding, PT, ROTEM, Biomarker

## Abstract

**Background:**

Fibrinogen and prothrombin have been suggested to become rate limiting in trauma associated coagulopathy. Administration of fibrinogen is now recommended, however, the importance of prothrombin to patient outcome is unknown.

**Methods:**

We have utilized two trauma patient databases (database 1 *n* = 358 and database 2 *n* = 331) to investigate the relationship of plasma prothrombin concentration on clinical outcome and coagulation status. Database 1 has been used to assess the relationship of plasma prothrombin to administered packed red blood cells (PRBC), clinical outcome and coagulation biomarkers (Prothrombin Time (PT), ROTEM EXTEM Coagulation Time (CT) and Maximum Clot Firmness (MCF)). ROC analyses have been performed to investigate the ability of admission coagulation biomarkers to predict low prothrombin concentration (database 1), massive transfusion and 24 h mortality (database 1 and 2). The importance of prothrombin was further investigated in vitro by PT and ROTEM assays in the presence of a prothrombin neutralizing monoclonal antibody and following step-wise dilution.

**Results:**

Patients who survived the first 24 h had higher admission prothrombin levels compared to those who died (94 vs.67 IU/dL). Patients with lower transfusion requirements within the first 24 h (≤10 units of PRBCs) also had higher admission prothrombin levels compared to patients with massive transfusion demands (>10 units of PRBCs) (95 vs.62 IU/dL). Admission PT, in comparison to admission ROTEM EXTEM CT and MCF, was found to be a better predictor of prothrombin concentration <60 IU/dL (AUC 0.94 in database 1), of massive transfusion (AUC 0.92 and 0.81 in database 1 and 2 respectively) and 24 h mortality (AUC 0.90 and 0.78 in database 1 and 2, respectively). In vitro experiments supported a critical role for prothrombin in coagulation and demonstrated that PT and ROTEM EXTEM CT are sensitive methods to measure low prothrombin concentration.

**Discussion:**

Our analyses suggest that prothrombin concentration at admission is predictive of mortality and transfusion and indicates that prothrombin and fibrinogen are rate limiting in coagulopathy.

**Conclusions:**

Admission PT is predictive of low prothrombin concentration and clinical outcome. PT could therefore be used as a surrogate for prothrombin concentration and further evaluation of point-of-care devices for faster PT analysis is warranted.

## Background

Hemorrhage is still a common cause of death in trauma. Even when patients are rapidly brought to hospital and are treated according to current trauma guidelines, patients may die because of bleeding that cannot be controlled [1, 2]. Coagulopathy is associated with worse outcomes and may develop during hemorrhage or transfusion therapy [3–5]. Fibrinogen and prothrombin have been suggested to be the first coagulation factors to become rate limiting for coagulation in such situations [6]. While the administration of fibrinogen is now recommended to restore fibrinogen levels [7–9], the importance of prothrombin in trauma-induced coagulopathy and patient outcome is unclear.

In experimental models of dilutional coagulopathy, administration of recombinant human (rh) prothrombin alone or in combination with fibrinogen improved survival, reduced blood loss and improved coagulation when measured by ROTEM and PT [10, 11]. Administration of prothrombin-containing concentrates may therefore be useful also in trauma patients with coagulopathy. Measurement of prothrombin concentration is necessary to identify patients with low levels and address the concern that over supplementation could lead to increased thrombotic risk [12–14]. However, since central lab analyses of plasma samples for prothrombin concentration are too slow, there is a need for surrogate biomarkers to identify patients with low prothrombin concentration. We have therefore conducted analyses of prothrombin levels in a prospective observational cohort from a London Trauma Centre and extended analyses on outcomes in an independent cohort from the Medical University of Innsbruck.

The aims of the study were, firstly, to investigate the consequence of admission prothrombin concentration on massive transfusion (more than 10 PRBC units) and mortality at 24 h. Secondly, to determine the relationship between admission biomarkers (PT, ROTEM EXTEM CT and MCF) and prothrombin concentration, and thirdly, to understand the ability of admission biomarkers to act as a surrogate for low prothrombin concentration and predict outcome. To further evaluate the individual role of prothrombin on PT and ROTEM biomarkers, in vitro studies were performed to investigate the effect of specific prothrombin depletion.

## Methods

### Analyses of prospective observational cohort studies

#### Databases

Database 1 (London) was generated from the Activation of Coagulation & Inflammation in Trauma (ACIT) study, a prospective observational cohort study conducted at the Royal London Hospital, London, UK. Patient samples used in this study were collected between January 2008 and September 2013. The ACIT study was reviewed and approved by the UK Regional Ethics Committee [15]. All adult trauma patients (>15 years) who met the local criteria for full trauma team activation were eligible for enrollment and recruited into the study when research personnel were present (08:00-00:00 daily). Exclusion criteria were: arrival in the emergency department 2 h after injury; the administration of 2000 mL of intravenous fluid before emergency department arrival; transfer from another hospital; and burns covering 5% of the total body surface area. Patients were retrospectively excluded if they declined to give consent to use research samples collected, were found to be taking anticoagulant medications, had moderate or severe liver disease or a known bleeding diathesis.

Database 2 (Innsbruck) was generated from the Diagnosis and Treatment of Trauma-Induced Coagulopathy (DIA-TRE-TIC) study, a single center prospective cohort study conducted at Innsbruck Medical University Hospital between July 2005 and July 2008. Adult poly-trauma patients, who were admitted to the Level I Trauma Center at Innsbruck Medical University Hospital were eligible for enrollment and recruitment into the study. Starting in January 2006, patients with isolated traumatic brain injury were enrolled as well. Severe poly-trauma was defined as an Injury Severity Score (ISS) of ≥15 resulting from injury of at least two body regions. Isolated head injury was defined as a Glasgow Coma Score of ≤14 after blunt head trauma in patients with an Abbreviated Injury Score (AIS) of <3 in any other body region. Exclusion criteria were: patients <18 years, penetrating injuries, admittance to the study hospital later than 12 h after trauma, pre-existing coagulopathy, burn injury, malignant disease, avalanche victims, or exhibition of non-head single trauma. The study protocol was approved by the Ethics Committee of Innsbruck Medical University. The need for written informed consent was waived because study-related blood sampling was judged a minimal-risk intervention and all patients were treated according to routine institutional treatment guidelines [16].

Data have been collected by each trauma center and locally available methods have been used for sample analyses. Prothrombin concentration for database 1 was measured in the hospitals central lab with a prothrombin clot assay and a pooled normal plasma standard calibrated to an international standard for prothrombin. In both hospitals ROTEM analyses were performed locally to the emergency department, whilst PT was analysed in the hospitals central lab. In order to evaluate the predictive ability of admission coagulation biomarker data under real conditions no attempt was made to monitor variation or standardize sample analyses between hospitals.

#### Outcome measures

The main outcomes considered were prothrombin concentration (IU/dL) measured at admission, total amount of PRBC (units) delivered during the first 24 h, and the proportion survival (%) at 24 h after admission. Three binary variants of these outcomes were also defined: low prothrombin (indicator of concentration <60 IU/dL at admission), massive transfusion (indicator of >10 PRBC units) and mortality at 24 h (indicator of death within the first 24 h). Biomarkers included in analyses were fibrinogen concentration, EXTEM CT, EXTEM MCF and PT, all measured at admission.

#### Statistical analyses

The relationship between prothrombin concentration at admission with survival at 24 h and, the amount PRBC delivered during the first 24 h after admission, as well as with fibrinogen concentration, EXTEM CT, EXTEM MCF and PT, was investigated graphically for database 1. Furthermore, Spearman’s correlation coefficient was used to quantify the relationships between prothrombin concentration and fibrinogen concentration, EXTEM CT, EXTEM MCF and PT values. Prothrombin and fibrinogen concentration, EXTEM CT, EXTEM MCF and PT are all admission values.

For each of the three binary outcomes the predictive ability of EXTEM CT, of EXTEM MCF and of PT was assessed using receiver operating characteristic (ROC) analyses. Comparison between EXTEM CT, EXTEM MCF and PT was quantified by the area under the ROC (AUC). DeLong’s test [17] was used to assess statistical difference between the AUCs. ROC analyses were performed using R package pROC [18]. Graphs and analyses were performed using R version 3.1.0 and above (R Foundation for Statistical Computing, Vienna, Austria). The significance level used was 0.05.

### In vitro prothrombin studies

#### Materials

The prothrombin neutralizing anti-human monoclonal antibody AB730006 ba SP14-046 IgG2b was obtained from MedImmune (Cambridge, UK).

Blood samples intended for in vitro studies at AstraZeneca R&D, Gothenburg, Sweden were drawn from 5 healthy volunteers (3 males and 2 females) after informed consent and approval from the local Gothenburg ethical committee (ethical permit number: 033-10). The sample size was not determined from a formal power calculation for this particular study but was based on previous experience of the assay methods used. Whole blood was collected into polypropylene tubes from Sarstedt (Nümbrecht, Germany) by free flow from a 17-gauge Venflon needle from Becton Dickinson Infusion Therapy AB (Helsingborg, Sweden). The first 2 mL of the collected blood was discarded. Then nine volumes blood was mixed with one volume 0.11 M trisodium citrate. Of the 50 mL whole blood collected per donor 10 mL was used for preparation of platelet poor plasma by centrifugation of the citrated blood at 2000 x *g* in a swing-out rotor for 20 min at 20 °C prior to transfer of the plasma supernatant to a new sample tube.

#### Rotational thromboelastometry

Clot elasticity was measured by a standard coagulation test (EXTEM + STARTEM reagents, Tem Innovations GmbH, Munich, Germany) using ROTEM equipment (Tem Innovations GmbH). Citrated blood, as described above, was pre-incubated with prothrombin neutralizing anti-human antibody (MedImmune) 0.021, 0.041, 0.062 and 0.083 mg/mL to study the effect of a specific decrease in prothrombin. A serial dilution was also performed to study the effect of a simultaneous decrease in all coagulation and anticoagulation factors. This step-wise dilution was done with 13 mM sodium citrate buffer starting at 100% whole blood and then 70, 50, 40, 30, 20, 10 and 5% whole blood. Samples were kept at 37 °C for 10 min before starting ROTEM analysis. Sodium citrate was included in the dilution buffer to keep the citrate concentration constant during dilution of the citrated whole blood. Thus, the free calcium concentration following addition of calcium for recalcifying purposes as part of the standard assay was kept constant. EXTEM CT and EXTEM MCF were evaluated by adding 300 μL of the incubated blood samples into the ROTEM equipment together with 20 μl EXTEM reagent and 20 μL STARTEM reagent according to the manufacturer instructions. All samples were run for 60 min.

#### Prothrombin time

Citrated plasma was pre-incubated with prothrombin neutralizing anti-human antibody (MedImmune) 0.021, 0.041, 0.062, 0.083, 0.124 and 0.165 mg/mL or diluted with 20 mM sodium citrate starting at 100% and then 70, 50, 40, 30, 20, 10 down to 5% plasma and kept at 37 °C for 9 min before PT was measured by adding 25 μL plasma to the KC 10 A micro coagulometer (Amelung, Lemgo, Germany). Dilution with a sodium citrate buffer for ROTEM analysis was performed as described for the diluted whole blood samples although the concentration of citrate had to be increased to compensate for the lack of hematocrit in plasma. After a 1 min incubation period at 37 °C in the coagulometer, 50 μL of Thromborel^®^ S (Siemens Healthcare GmbH, Marburg, Germany) was added and the time to coagulation was measured. Maximum measurement time was 15 min.

### Plasma concentration of prothrombin

Prothrombin functional activity was measured in the antibody-treated and diluted blood/plasma samples using a chromogenic prothrombinase end-point method (PI 200040 Rox Prothrombin, Rossix AB, Mölndal, Sweden) and a SPECTRAmax plate reader (Molecular Devices, California, USA). As a standard a recombinant prothrombin preparation calibrated to the NIBSC 98/590 standard was used. One mg prothrombin was found to be equivalent to 8.74 IU. This information was used to calculate IU/dL from the mg/mL result obtained with the prothrombinase assay.

## Results

### Association of prothrombin concentration with mortality and transfusion demand in patients

Data including survival, administered PRBC and admission PT, EXTEM CT and MCF were available from 358 patients from London (database 1) and 331 patients from Innsbruck (database 2) (Table 1). Prothrombin was not a routine biomarker for analysis at the Medical University of Innsbruck and therefore the number of patients with admission data was limited. Database 2 was therefore only used where analyses was independent of prothrombin.

**Table 1:** Patient characteristics at admission to emergency department, or within 24 h of admission (24 h survival, and ≥1 PRBC). Data shown is median (IQR) unless otherwise stated

	Database 1 London *n* = 358^a^	Database 2 Innsbruck *n* = 331^b^
Men, *n* (%)	288 (80%)	259 (78%)
Age, years	35 (23–50)	43 (27–56)
Time from injury, minutes	76 (58–101)	75 (55–120)
Glasgow Coma Score (GCS)	15 (14, 15)	11 (6–15)
Injury Severity Score	13 (5–27)	34 (24–45)
Systolic BP, mmHg	132 (110–150)	120 (100–140)
Temp, °C	35.8 (35.1–36.5)	35.0 (34.2–36.0)
Hct	0.4 (0.37–0.43)	0.34 (0.28–0.38)
Platelets, 10^9^/L	233 (194–273)	170 (139–203)
Hb, g/dL	13.8 (12.5–14.9)	11.6 (9.5–13.1)
AT, IU/dL	94 (82–104)	68 (56–81)
Factor VII, IU/dL	94 (76–118)	83 (50–98)
Factor X, IU/dL	96 (82–112)	69 (46–88)
24 h survival, *n* (%)	347, (97%)	308, (92%)
≥1 PRBC units, *n* (%)	104 (29%)	148 (44%)
Prothrombin, IU/dL	93 (77–105)	68 (55–86)
Fibrinogen, g/L	2.2 (1.7–2.7)	2.1 (1.6–2.6)
EXTEM CT, seconds	62 (50–77)	63 (55–74)
EXTEM MCF, mm	60 (56–63)	52 (46–57)
PT, seconds	11.1 (10.6–11.8)	14.9 (13.1–17.0)

^a^At most 6% missing values in any variable except for Time from injury, Temp, and GCS which had 10, 36 and 44% missing values respectively

^b^Factor VII, Factor X and Prothrombin were missing in 86% of the subjects, Time from injury and Temp in 20 and 62%, respectively. The rest of the variables had missing values in at most 4% of the 331 subjects

The characteristics of the two populations differed in injury severity score (ISS: median 13 and 34 in database 1 and 2 respectively), percentage of patients receiving one or more PRBC units (29 and 44%), and also in age, temperature, systolic blood pressure and platelets (Table 1).

**Fig. 1:**
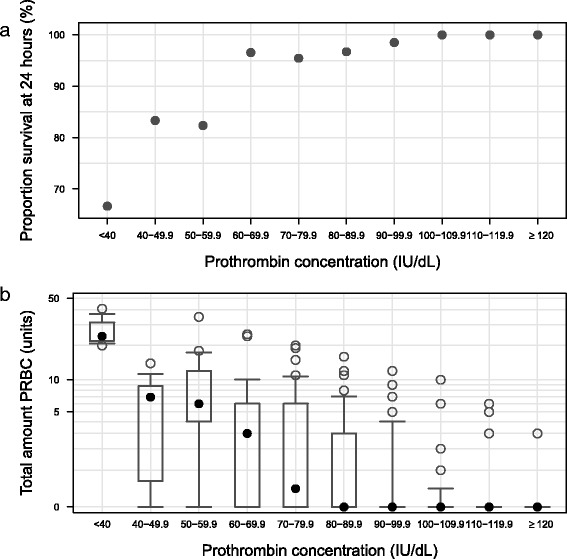
24 h survival **a** and amount of PRBC delivered during the first 24 h after admission **b** are plotted versus prothrombin concentration at admission for database 1. For clarity prothrombin concentration is divided into intervals (n_interval_ = (3, 6, 17, 29, 44, 61, 67, 66, 32, 33)). In the *upper graph* **a** proportion of 24 h survival is plotted versus prothrombin concentration interval, and in the *lower graph* **b** distribution of units of PRBC delivered during the first 24 h is plotted versus prothrombin concentration interval using box plots (*box* representing 25^th^, median (*dot*) and 75^th^ percentiles, whiskers go out to 10^th^ and 90^th^ percentiles). Note: only 11 out of the 358 patients included in the graph died within 24 h

Survival at 24 h was associated with higher prothrombin plasma concentrations at admission with a change in the proportion of survivors occurring within the range of 50–70 IU/dl (Fig. 1a). A total of 55, 26, and 9 patients had admission prothrombin plasma concentration below 70, 60 and 50 IU/dl respectively, and the corresponding surviving percentages at 24 h were 89, 81 and 78%. In database 1, patients who survived the first 24 h had a median prothrombin concentration of 94 IU/dL compared to 67 IU/dL for those who did not survive. Patients with an increased transfusion demand (units of PRBC given within the first 24 h) also had a lower prothrombin concentration at admission (Fig. 1b). Patients receiving ≤10 units PRBC during the first 24 h had a median prothrombin concentration of 95 IU/dL compared to 62 IU/dL for those who received >10 units.

### Impact of prothrombin concentration on EXTEM CT, EXTEM MCF and PT in vitro

**Fig. 2:**
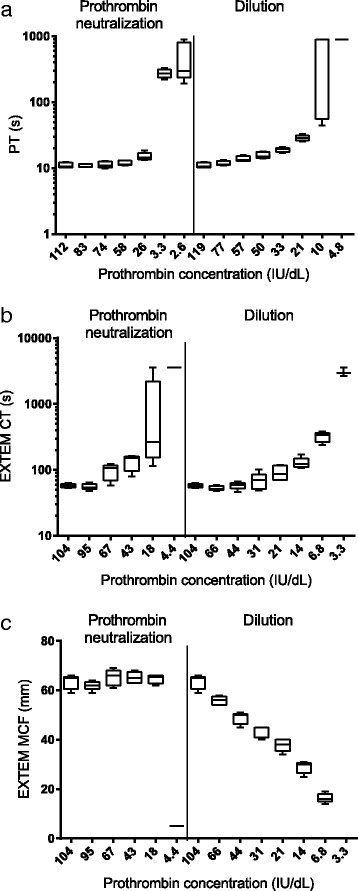
Impact of prothrombin depletion by adding increasing amount of neutralizing antibody or step-wise dilution for **a** Prothrombin time (PT), **b** ROTEM EXTEM Coagulation time (CT) and **c** ROTEM EXTEM Maximum Clot Firmness (MCF). The PT experiment was performed in citrated plasma from 5 different donors and the ROTEM EXTEM experiments were performed in citrated whole blood from the same 5 donors. The boxes represent 25^th^ to 75^th^ percentiles, the *horizontal bar* in the box is median and the whiskers are min and max values

To further investigate the importance of prothrombin concentration on coagulation we performed in vitro experiments in blood or citrated plasma from five healthy volunteers in which the prothrombin concentration was either gradually reduced by step-wise dilution or by the addition of increasing amounts of a monoclonal antibody neutralizing the prothrombin activity. Specific inhibition or dilution reduced the prothrombin concentration and had a dramatic effect on coagulation when measured by PT or EXTEM CT. With the PT assay, the transition point for a prolonged coagulation time occurred at similar concentrations, approximately 50–58 IU/dL prothrombin (Fig. 2a). The EXTEM CT was first prolonged at approximately 67 IU/dL prothrombin with the neutralizing antibody added, but only at approximately 44 IU/dL when the blood was diluted (Fig. 2b). EXTEM MCF showed no decrease when only prothrombin was increasingly neutralized until very low concentrations remained at which point it dropped dramatically (Fig. 2c). This was in contrast to the almost linear decline when the blood sample was step-wise diluted.

### Association of admission prothrombin concentration with PT or EXTEM CT and EXTEM MCF in patients

**Fig. 3:**
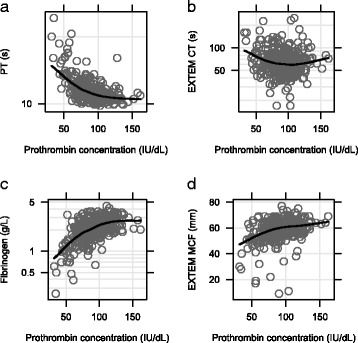
Prothrombin Time (PT) **a** EXTEM Coagulation Time (CT) **b** Fibrinogen **c** and EXTEM Maximum Clot Firmness (MCF) **d** versus prothrombin concentration for database 1. Loess curves (local regression curves) are added to aid in evaluating the relationship between variables. Fibrinogen concentration, CT and PT were log transformed due to skewed distributions

To investigate if currently available biomarker assays could be used to identify patients with low prothrombin plasma concentration we examined how admission prothrombin concentration correlated with admission fibrinogen concentration, EXTEM CT, EXTEM MCF and PT in database 1. There was a significant correlation between prothrombin concentration with fibrinogen concentration and all coagulation endpoints tested (Fig. 3a–d). Spearman’s correlations for PT and prothrombin were −0.56, *p*-value < 0.001, for CT and prothrombin −0.11, *p*-value = 0.04, for fibrinogen and prothrombin 0.55, *p*-value < 0.001 and finally for MCF and prothrombin 0.35, *p*-value < 0.001.

### Assessment of PT, EXTEM MCF and EXTEM CT as predictors of prothrombin concentration and outcome in patients

**Fig. 4:**
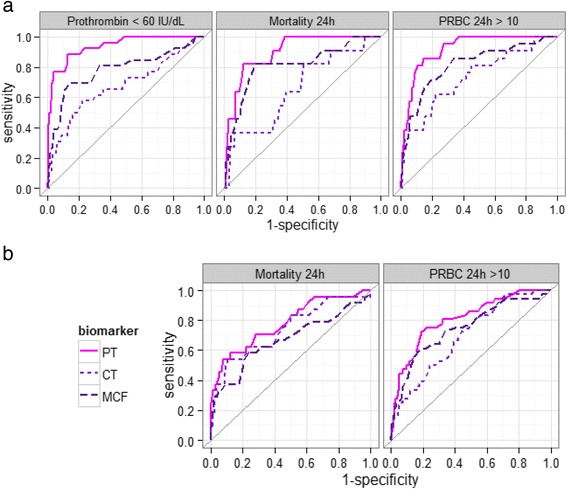
ROCs for each of PT, EXTEM Maximum Clot Firmness (MCF) and EXTEM Coagulation Time (CT), when entered as single predictor of each outcome indicator in the underlying logistic regression. **a** ROCs based on database 1. **b** ROCs based on database 2

The ability of PT, EXTEM MCF and EXTEM CT biomarkers to predict the presence of low prothrombin concentration (<60 IU/dL at admission), massive transfusion (>10 PRBC units within the first 24 h) and mortality within the first 24 h were compared using ROC analyses. The AUC of a ROC provides an assessment of the overall value of the biomarker as a predictor. Based on database 1, the AUCs of PT, of EXTEM CT and of EXTEM MCF were 0.94, 0.68, and 0.78 respectively, for prediction of low prothrombin concentration (Fig. 4a left panel, Table 2). The AUC of PT differed significantly from the AUC of EXTEM MCF, and from the AUC of EXTEM CT (*p* <0.001 for both comparisons).

**Table 2:** Summary of ROC analyses

Database	Biomarker	Low prothrombin		Mortality 24 h		Massive transfusion	
		AUC (95% CI) *p*-value		AUC (95% CI)	*p*-value	AUC (95% CI)	*p*-value
1 *n* = 358	PT	0.94 (0.88,0.96)	–	0.90 (0.82,0.97)	–	0.92 (0.89,0.98)	–
	EXTEM CT	0.68 (0.61,0.85)	<0.001	0.66 (0.48, 0.82)	<0.001	0.73 (0.55,0.80)	<0.001
	EXTEM MCF	0.78 (0.71,0.92)	0.003	0.81 (0.66,0.96)	0.04	0.81 (0.67,0.89)	0.04
2 *n* = 331	PT	Not applicable		0.78 (0.68,0.89)	–	0.81 (0.73,0.89)	–
	EXTEM CT			0.74 (0.62,0.86)	0.44	0.70 (0.61,0.78)	0.008
	EXTEM MCF			0.67 (0.54,0.81)	0.03	0.75 (0.66,0.84)	0.05

The area under the ROC (AUC), was calculated in separate logistic regression models for each of the binary response variables low prothrombin (<60 IU/dL), mortality 24 h and massive transfusion (PRBC 24 h >10 units) for each of the three biomarkers PT, EXTEM CT and EXTEM MCF. The AUC *p*-value refers to a two-sided test of the null hypothesis of no difference in AUC between PT and each of the two other biomarkers. In database 2 there were too few patients with admission prothrombin concentrations to perform a valid ROC analysis

In order to complete the linkage between PT as a surrogate biomarker for prothrombin concentration, ROC analyses were performed to investigate the ability for PT to predict survival and transfusion demand. Based on database 1, PT was a significantly better predictor of 24 h mortality (AUC 0.90) and of massive transfusion (AUC 0.92) than EXTEM CT or EXTEM MCF (Fig. 4a and Table 2). In database 2, PT was the better predictor and highest AUC for both endpoints, however, the difference between PT and the two other biomarkers was smaller for database 2 (Fig. 4b and Table 2).

.

## Discussion

In this study we investigated the critical role of prothrombin concentration in coagulopathy and prediction of outcome in coagulopathic trauma patients. In addition, we evaluated currently available coagulation biomarkers as surrogates for detecting critically low plasma prothrombin levels. Data presented indicates that admission prothrombin plasma concentration can be used to predict increased survival and a lower transfusion demand within the first 24 h following admission of the trauma patient and that a cut-off in the range of 50–70 IU/dL at admission is associated with a worse outcome (Fig. 1). During such a coagulopathy many coagulation factors are simultaneously reduced. This has been previously shown where coagulation factors and biomarkers were analysed in coagulopathic patients with INRs of 1.5–3 prior and post FFP administration [19]. Similar to our findings, these patients had a median prothrombin concentration of 34% of normal before FFP administration and also reduced concentrations of several other coagulation factors such as Factor X and fibrinogen.

Since several coagulation factors are reduced in coagulopathic trauma patients we investigated the specific role of prothrombin concentration in coagulation by in vitro experiments. Our in vitro data comparing specific neutralization of prothrombin by a prothrombin neutralizing antibody and general dilution of all coagulation factors by serial dilution of blood or plasma, showed that reduction in prothrombin concentration had a dramatic effect on both PT and ROTEM CT. Comparable data was found for specific neutralization of prothrombin or general dilution of all coagulation factors suggesting that prothrombin concentration is a rate limiting factor in coagulation (Fig. 2). Our in vitro results also support the usefulness of PT and EXTEM CT as surrogate markers for direct measurement of prothrombin concentration. EXTEM MCF is not appropriate due to the discrepancy between ROTEM MCF obtained during step-wise dilution and neutralization of prothrombin. This is thought to be due to that following neutralization a proportion of prothrombin is still activated and is available to activate platelets and fibrinogen which are at normal concentration. Since fibrinogen and platelets are mainly responsible for building MCF the same clot stability is reached eventually. In the case of step-wise dilution, all coagulation factor concentrations and platelet count are decreased and hence the almost linear decrease in EXTEM MCF.

Extending in vitro findings to the trauma databases, prothrombin concentration in trauma patients was also reflected in the biomarkers PT, EXTEM CT and EXTEM MCF. Furthermore, low prothrombin concentration was correlated with a low fibrinogen concentration (Fig. 3). This suggests that the trauma patients having low fibrinogen and prothrombin concentrations are coagulopathic due to coagulation factor consumption and/or dilution. Since point-of-care (PoC) tests are not available for the fast analysis of fibrinogen or prothrombin, surrogate coagulation markers could be used to identify patients with low prothrombin concentrations. The idea of early identification of patients in need of hemostatic therapy by the use of coagulation assays is not new. ROTEM and TEG, where available, are used for this purpose and for guiding therapy [7–9, 20]. In hemorrhagic patients the PT assay has as far as we are aware been used to analyse retrospective data, although it is sometimes used as a tool to confirm the effectiveness of hemostatic therapy [21].

In the clinic, a cut-off value to define “low prothrombin” is required in order to decide who will receive prothrombin containing treatment and who will not. A cut-off value for prothrombin is also needed for ROC analysis in order to compare the sensitivity and specificity of biomarkers to predict low prothrombin concentration. From Fig. 1, we observed that there appears to be a change in the proportion of survivors and the total amount of administered PBRC within the prothrombin concentration range of 50–70 IU/dL. Furthermore, in vitro data presented in Fig. 2 shows that decreasing prothrombin below a similar limit has a large impact on PT and EXTEM CT. These two observations together provides evidence for a threshold in prothrombin concentration within the range of 50–70 IU/dL that is critical for coagulation. In order to evaluate biomarker predictivity we chose to evaluate 60 IU/dL as a binary threshold for ROC analyses since a prothrombin concentration below 60 IU/dL is considered to be out of the normal range and therefore is reflective of a clinically relevant cut-off.

ROC analyses suggests that PT is a better predictor of low prothrombin concentration versus EXTEM CT and EXTEM MCF. To check how sensitive the biomarker comparisons were to the choice of cut off level, we also performed the analyses using < 70 IU/dL. The ranking of the coagulation biomarkers remained the same independently of whether the cut off was set to < 60 (Fig. 4) or < 70 IU/dL (data not shown). Furthermore, PT was also the better predictor of massive transfusion and mortality compared to EXTEM CT and EXTEM MCF. We chose not to include the amplitudes at 5 or 10 min (A5, A10) in our analyses since they have been shown to correlate well to MCF [22–24]. In the databases analysed even a small increase in PT is associated with an increased risk. This was observed in both independent databases despite multiple differences in the severity of injury and inclusion/exclusion criteria.

We believe that successful normalization of prothrombin levels requires administration of a concentrate such as the recombinant human prothrombin (MEDI8111) that was recently evaluated in healthy volunteers [25]. In support of this, administration of recombinant human prothrombin alone was sufficient to reduce bleeding and improve coagulation and survival time in coagulopathic pigs [11]. In humans, however, the importance of having a sufficiently high fibrinogen concentration has been recognized [7] and from our database analyses we confirm that there is a strong correlation between admission concentrations of fibrinogen and prothrombin (Fig. 3). We cannot therefore distinguish between the specific contribution of fibrinogen or prothrombin loss alone on prolonged PT and coagulopathy but instead conclude that prothrombin is depleted along with fibrinogen during the development of coagulopathy and that replacement of both factors is required to achieve hemostasis.

PT is a useful biomarker to identify eligible patients for prothrombin replacement therapies although centrally measured PT was used in the current databases which is recognised to have limitations in guiding treatment due to the slow turnaround time [26]. A number of PoC analysers of PT are available e.g. CoaguChek XS system, Roche; i-STAT system, Abbott, and need to be evaluated further for this purpose. If this can be achieved, it seems possible that in the future massive bleedings can be controlled or even avoided by identification of coagulopathic patients depleted in prothrombin by simple available PoC methodology.

## Limitations

This study is based on analyses of two prospective observational cohorts. Since prothrombin concentration was not routinely measured at the Medical University of Innsbruck the number of patients with admission data was limited. All analyses involving prothrombin were therefore restricted to database 1 but database 2 was included and added value by extending observations regarding the predictivity of PT for mortality and transfusion in two independent databases. There are also multiple differences in the databases with regards to severity of injury, inclusion/exclusion criteria, biomarker analyses and importantly admission sampling time from injury. While this variability does add noise to the study we consider that it also adds strength in our conclusions i.e. that despite these differences, PT is still predictive of outcome and the better biomarker in both databases and as a consequence supportive of broader clinical utility.

## Conclusions

From this study we conclude that low prothrombin concentration with a cut-off in the range of 50–70 IU/dL (corresponding to approximately 50–70% of normal prothrombin concentration) together with low fibrinogen is rate limiting for coagulation in bleeding trauma patients and is associated with an increased risk for massive transfusion and mortality. PT at admission is a sensitive and specific predictor of low prothrombin concentration and is a better predictor of massive transfusion and mortality than the ROTEM parameters EXTEM CT and EXTEM MCF used for current guidance.

## Abbreviations

CT: Coagulation time
FFP: Fresh frozen plasma
INR: International Normalized Ratio
ISS: Injury severity score
MCF: Maximum clot firmness
PoC: Point of care
PRBC: Packed red blood cells
PT: Prothrombin time
ROC: Receiver operating characteristic (curve)
ROTEM: Rotational thromboelastometry
TEG: Thromboelastography
